# Impact of *Chlorella vulgaris* powder on the nutritional content and preference of Khalas date spread

**DOI:** 10.3389/fnut.2025.1617754

**Published:** 2025-08-13

**Authors:** Rehab F. M. Ali, Ayman M. El-Anany, Mona S. Almujaydil, Raghad M. Alhomaid, Hend F. Alharbi, Reham M. Algheshairy, Nada A. Alzunaidy, Seham S. Alqaraawi

**Affiliations:** ^1^Department of Food Science and Human Nutrition, College of Agriculture and Food, Qassim University, Buraydah, Saudi Arabia; ^2^Department of Special Food and Nutrition, Food Technology Research Institute, Agricultural Research Center, Giza, Egypt

**Keywords:** antioxidants, amino acids, diet, diet supplementation, date spread

## Abstract

**Introduction:**

The current study aimed to investigate the nutritional and sensory benefits of date fruit spreads formulated with Khalas date, olive oil, psyllium, roasted coffee, cocoa powder (CP), and incorporated with different quantities of *Chlorella vulgaris* powder (CVP).

**Methods:**

The original spread consists of 65% Khalas dates, 30% Extra virgin olive oil (EVOO), and smaller amounts of psyllium, coffee, cocoa powder, and salt. By substituting EVOO with CVP in different proportions, the healthy spread formulas were produced.

**Results and discussion:**

Protein content increases from 2.21% in control to 9.82% with 10% CVP. Ash content rises significantly with 10% CVP, with values up to 3.04 times higher than control. A 10% addition of CVP aligns the amino acid profile with FAO/WHO standards, except for lysine, which reaches 65.37% of the recommended levels. Additionally, the polyunsaturated fatty acid content increases substantially, with linoleic acid rising from 5.08% to 6.45% and linolenic acid from 0.7% to 2.75% at the 10% CVP level. DPPH radical scavenging activity, improves significantly, indicating the potential of CVP as a functional food ingredient. While lower concentrations of CVP (2-4%) do not significantly affect acceptability, higher concentrations (6-10%) lead to significant declines in consumer ratings.

## 1 Introduction

Governments worldwide have adapted their dietary recommendations to combat nutrient deficiencies and chronic diseases, leading to the establishment of Dietary Reference Intakes (DRIs) and Food-Based Dietary Guidelines (FBDG). These frameworks are tailored to local dietary habits and health needs, with innovative approaches like Japan’s Optimized Nutri-Dense Meals enhancing public understanding of nutrition (1). Additionally, International strategies aimed at reducing nutrients such as saturated fats and sugars are crucial for improving population health. These strategies, often endorsed by the World Health Organization (WHO), focus on reformulating food products and implementing public health policies to mitigate the risks associated with non-communicable diseases (NCDs) (2–4). The integration of nutrition science into public health policies reflects a shift towards holistic approaches that consider physiological and psychological factors in dietary recommendations (5, 6). Each food group supplies a distinct set of nutrients and bioactive substances to the diet. At the same time, no single group can provide all of the critical nutrients required for optimal health. According to Comerford et al. (7), achieving nutritional sufficiency requires a varied range of foods. Briefly, dietary groups complement each other rather than being interchangeable. Each food group contributes different nutrients and bioactive substances to a healthy diet. At the same time, each group cannot offer all of the nutrients essential for optimal health. Food categories are not equivalent, but rather complementary. Date palm fruit (*Phoenix dactylifera* L.) is recognized for its extensive nutritional and health benefits, making it a valuable addition to a healthy diet. Dates are high in carbohydrates, primarily sugars like glucose and fructose, providing a quick energy source (8). They are also rich in dietary fiber, which aids digestion and promotes gut health (2, 9). Minerals found in dates include potassium, magnesium, and calcium, contributing to overall health (10). Dates possess antioxidant, antibacterial, and anti-inflammatory properties, which may help in preventing chronic diseases such as diabetes and cancer (9, 10). They have been shown to improve treatment outcomes in pediatric cancer patients and may protect against conditions like ulcerative colitis (10). The fruit’s high fiber content can assist in weight management and appetite control (8). The production of dates has evolved significantly, with modern technologies enhancing the utilization of surplus and second-class dates. These advancements have led to the creation of various derived products, such as date syrup and paste (11). The fermentation of date sugars can yield vinegar, while sugar extraction processes have been optimized to recover high sugar content from date waste (12). Dates serve as a natural sweetener alternative, reducing reliance on synthetic sugars and contributing to healthier food options (13). Dietary fat plays a crucial role in health, polyunsaturated fatty acids (PUFAs) and monounsaturated fatty acids (MUFAs) are associated with lower risk of heart disease mortality (14), improved lipid profiles and metabolic health (15), MUFAs has been shown to lower LDL cholesterol levels and improve lipid profiles, which is crucial for cardiovascular disease prevention (16), anti-inflammatory properties that can improve insulin sensitivity, thereby aiding in the management of metabolic syndrome and obesity-related conditions (17). In this regard, Extra virgin olive oil (EVOO) is recognized for its beneficial effects on lipid profiles and cardiovascular health, primarily due to its rich content of monounsaturated fatty acids (MUFA) and phenolic compounds. These components contribute to the modulation of various cardiovascular risk factors, making EVOO a key element in dietary strategies aimed at improving heart health. EVOO consumption has been linked to reductions in low-density lipoprotein cholesterol (LDL-C) and total cholesterol levels, which are critical for cardiovascular health (18). The phenolic compounds in EVOO, such as hydroxytyrosol, exhibit strong antioxidant properties that help prevent atherosclerosis and endothelial dysfunction (19). Incorporating EVOO into a Mediterranean diet enhances its cardioprotective effects, as this diet is associated with lower incidences of cardiovascular diseases (20). Studies have shown that diets high in EVOO lead to significant improvements in various cardiometabolic markers, including reductions in glucose and inflammatory markers (18). Algae are increasingly recognized as a sustainable alternative protein source, offering a rich nutritional profile that includes proteins, essential fatty acids, vitamins, and minerals. Their potential as food additives is underscored by their ability to enhance the nutritional value of various products while addressing environmental concerns associated with traditional protein sources. Algae can contain up to 70% protein by dry weight, surpassing traditional sources like meat and legumes (21). They are rich in essential amino acids, long-chain polyunsaturated fatty acids, carotenoids, and phenolics, contributing to overall health (22). Algal proteins have favorable amino acid profiles, making them suitable for dietary supplementation and food formulation (23). Algae require minimal freshwater and land, thriving in non-arable environments, thus reducing agricultural pressure (23). They absorb CO2, contributing to climate change mitigation while producing biomass (21). Sensory attributes such as taste and odor can hinder acceptance, thus, improving these aspects is crucial (22). The recent emphasis on healthier food spreads has spurred innovative formulations that prioritize nutritional value and sustainability. Various studies highlight the development of spreads that incorporate underutilized ingredients, functional components, and improved nutritional profiles, catering to the growing consumer demand for healthier options. Peanut and Baru Almond Spread was developed using baru almonds and ora-pro-nóbis mucilage, resulting in a 16% increase in protein content and enhanced antioxidant activity compared to traditional peanut butter (24).

Sweet spreads, including jams and nut-based options, have also gained popularity, reflecting a broader trend towards plant-based diets (25). Strawberry Spreadable Cream utilized regional ingredients, promoting sustainability and reducing food waste, while offering nutritional advantages over existing market options (26). Fruit spreads can play a significant role in addressing malnutrition and enhancing nutrient intake, particularly among vulnerable populations. These nutrient-dense products are designed to deliver essential vitamins and minerals, making them a practical solution for improving dietary quality (27). They can effectively prevent micronutrient deficiencies, especially in children, by incorporating fruits known for their high biological activity and safety (28). These spreads are easy to consume and can be integrated into various diets, making them appealing to children and other high-risk groups (29). Fruit spreads are particularly beneficial for malnourished children, as they can be consumed without preparation and are resistant to bacterial contamination (30). The adaptability of spread formulations allows for the inclusion of diverse ingredients tailored to specific nutritional needs, enhancing their effectiveness in different settings (31). The current study aimed to assess the nutritional, phytochemical, antioxidant, and sensory properties of date fruit spreads formulated from, Khalas date, extra virgin olive oil, Psyllium, light roasted coffee, unsweetened natural cocoa powder (CP), and incorporated with different quantities of *Chlorella vulgaris* powder (CVP).

## 2 Materials and methods

Khalas date, which is popular in Saudi Arabia, was chosen for this experiment. Khalas variety (production season 1,446 H, 2024). All experiments were conducted using the same batches of dates. Ten kilograms of Khalas Dates (*Phoenix dactylifera* L.) paste (KDP) were purchased from a local market in the city of Buraydah, Qassim, Saudi Arabia. Organic Extra Virgin Olive Oil (OEVOO), the production date October 2024, (with acid value 0.50 mg KOH.g-1, peroxide value 1.15 meq O2.kg-1) was obtained from Al-Jouf Agricultural Development Company in Saudi Arabia. Food grade *Chlorella vulgaris* powder (CVP) was obtained from Zhengzhou Sigma Chemical Co., Ltd. (Zhengzhou, China). Psyllium husk powder (PHP) was purchased from Imtenan Health Section, Food Additives Company, Egypt. Light roasted Coffee was purchased from a private Coffee producer, Buraidah city, Qassim, Saudi Arabia. Unsweetened natural cocoa powder was obtained from Kasih group for food products, Zarqa, Jordan. Salt was purchased from local market Buraidah city, Qassim, Saudi Arabia. The current investigation utilized analytical-grade chemicals and reagents from various commercial providers (Merck, Panreac, Scharlau, and Sigma-Aldrich).

### 2.1 Preparation of date fruit spreads

The original spread formula consists of Khalas dates (65%), extra virgin olive oil (EVOO, 30%), Psyllium (0.5%), light roasted Coffee (2%), unsweetened natural cocoa powder (2%) and salt (0.5%). Five formulas were prepared by partial replacement of EVOO in the original formula with proportions of 2, 4, 6, 8 and 10% of CV powder (Table 1). The formulated fruit spreads were individually homogenized, filled into sterilized glass jars (200 mL) then tightly closed and stored at refrigerated temperature (4°C) for further analysis. Each date spread formula consistes of 2 Kgs. Date spread formulas were prepared based on preliminary evaluation tests (unpublished data).

**Table 1:** Formulation of date spread with different levels of CV powder.

Ingredients	KDO0	KDO2	KDO4	KDO6	KDO8	KDO10
KDP	65	65	65	65	65	65
OEVOO	30	28	26	24	22	20
CVP	0	2	4	6	8	10
Psyllium husk (condensed)	0.5	0.5	0.5	0.5	0.5	0.5
Light roasted Coffee	2.0	2.0	2.0	2.0	2.0	2.0
Unsweetened natural cocoa powder	2.0	2.0	2.0	2.0	2.0	2.0
Salt	0.5	0.5	0.5	0.5	0.5	0.5

KDO0: control date spread formula (without CVP), KDO2: date spread fortified with 2% CVP, KDO4: date spread fortified with 4% CVP, KDO6: date spread fortified with 6% CVP, KDO8: date spread fortified with 8% CVP and KDO10: date spread fortified with 10% CVP.

### 2.2 Determination of proximate composition

The moisture content of samples was measured by drying at 105°C using AOAC method 925.09 (32). The Kjeldahl Technique (Method No. 978.04) (32) was used to determine total protein content (N × 6.25). In order to determinate crude fat, a known sample weight was extracted with petroleum ether (boiling point 60°C) using a Soxhlet apparatus (Method No. 930.09) (32). Ash content was measured by incineration (550°C) of known sample weights in a muffle furnace (Method No. 930.05) (32). Crude fiber was measured by digesting a known weight of fat-free sample in 1.25% sulfuric acid and 1.25% sodium hydroxide (Method No. 930.10) (32). The carbohydrate content was calculated by the differential. The percentages of moisture, fat, crude protein, ash, and crude fiber were subtracted from 100% (2). The Atwater conversion factor was used to calculate the energy content of samples based on their macronutrient composition. Atwater’s factors assign 9 kcal/g for lipids, and 4 kcal/g for both proteins and carbohydrates (33).

Minerals which include Ca, Fe, Zn, K, Cu, P, Mg, and Na were measured in a dilute solution of ashed samples using an atomic absorption spectrophotometer (3,300 Perkin-Elmer, SpectraLab Scientific Inc. 38 McPherson St. Markham, ON, CanadaL3R 3 V6), as reported by Ali (2).

### 2.3 Amino acid determination and P-PER

The amino acid (AA) profile and biological values of date spread samples were assayed as described by El Anany (34), which utilize hydrochloric acid to release amino acids for analysis. Alkaline Hydrolysis was used for tryptophan determination, following Miller’s techniques, which preserves this amino acid during analysis Miller’s (83). WHO/FAO (35) report was used to emphasized the need for adequate intake of indispensable amino acids (IAAs) to prevent deficiencies, particularly in developing countries (34). The predicted protein efficiency ratio (P-PER) formula, which incorporates leucine and tyrosine concentrations, was used to determine protein quality.

predicted protein efficiency ratio (P-PER) (2).


P−PER=−0.468+0.454leucine–0.105tyrosine.

### 2.4 Fatty acid analysis

The fatty acid composition of the samples was evaluated using a gas chromatograph with split/spitless injector and flame ionization detector (GC-FID, Dani Master GC, Dani Instrument, Milan, Italy). The instrument used a ZB-Wax column (Phenomenex, Torrance, CA, USA) with a 30 m length, 0.25 mm internal diameter, and 0.25 μm film thickness. The column oven temperature changed from 50°C (hold time 2 min) to 240°C (hold time 15 min), with a heating rate of 3°C/min. Injector and detector temperatures were set to 240°C. Helium gas was employed as the carrier, with a constant linear velocity of 30 cm/s. The results were expressed as relative area percentages (84).

### 2.5 Total phenolic, total flavonoids and antioxidant activity DPPH

The total phenolic content, total flavonoid content, and antioxidant activity (DPPH technique) of date spread samples were measured according to the methodologies reported by Alshammai et al. (36).

### 2.6 Sensory evaluation

To assess the sensory evaluation of produced date fruit spread (DFS) samples, 50 semi-trained panelists (aged 20–50 years) were randomly selected from staff members of a private date plant in Buraydah, Saudi Arabia, ensuring a diverse representation from the university community, using a ten-point hedonic scale. Panelists were allocated chairs in separate sets. All responders have given their agreement to participate in the study. Before the study started, the panelists provided informed, written permission. Panelists were invited to participate in a sensory evaluation training session for Check-All-That-Apply (CATA) products. Panelists rated samples individually, using water to cleanse their palates, which is crucial for minimizing bias in sensory assessments. Samples were evaluated on a ten-point hedonic scale (10 - greatly like to 1 - strongly detest) for appearance, flavor, taste, texture, color, and overall acceptability. The sensory evaluation met the requirements for sensory research established by the Auckland University of Technology Study’s Ethics Committee (AUTEC ethics application 16/340), ensuring the integrity of the research process.

### 2.7 Statistical analysis

SPSS (version 20) was implemented to analyze the data. The data were statistically analysed in five repetitions, excluding the sensory evaluation results (*n* = 50). The data were subjected to analysis of variance, and the means were separated using Duncan’s Multiple Range Test at 95% confidence.

## 3 Results and discussion

### 3.1 Chemical composition (g/100 g dry weight basis) and mineral content (mg/100 g) of date fruit spreads incorporated with different quantities of *Chlorella vulgaris* powder (CVP)

Table 2 shows the chemical composition (g/100 g dry weight basis) and mineral content (mg/100 g) of date fruit spreads incorporated with different quantities of CVP. Moisture content in date fruit spread formulas ranged from 12.00 to 13.98%, (Table 2). The incorporation of CVP into date fruit spreads significantly affects their chemical composition and moisture content, which contributes to extending the shelf life of these products. Lower moisture levels in formulations with CVP, compared to the control, enhance preservation by reducing microbial growth and enzymatic activity, thus prolonging shelf life. Reduced moisture levels inhibit spoilage, allowing for a shelf life exceeding 12 months at room temperature (37). The fat content in date fruit spread formulations varies significantly, with control samples containing extra virgin olive oil (EVOO) showing the highest fat level at 36.65%, indicating the richness of this oil in the spread. In contrast, spreads incorporating CVP exhibit lower fat concentrations, highlighting the impact of ingredient substitution on fat content (Table 2). Date spreads with CVP showed reduced fat levels, suggesting that the microalga may replace some of the fat content while still providing nutritional benefits (38). The use of CVP in date spreads may appeal to health-conscious consumers seeking lower-fat options without sacrificing nutrition (37). This microalga is rich in essential fatty acids, proteins, vitamins, and minerals, making it an attractive ingredient for enhancing the nutritional profile of food products without significantly increasing fat content (39, 40). CVP contains approximately 35–40% polyunsaturated fatty acids, including essential omega-3 and omega-6 fatty acids (39). CVP can be incorporated into various food products, including spreads, smoothies, and snacks, enhancing their nutritional value (38). The incorporation of CVP into date fruit spread significantly affects the ash content, with values ranging from 1.95% in the control to higher levels in CVP-enriched samples. This increase in ash content can be attributed to the mineral composition of CVP, which enhances the nutritional profile of the spread (Table 2). Ash content increased significantly, with values of 3.04, 2.64, 2.41, 1.68, and 1.46 times greater than the control for those samples incorporated with 2, 4, 6, 8, and 10% CVP, respectively. Higher ash content in food products often correlates with increased mineral availability, potentially enhancing the nutritional value of the date spread. Ash from biomass, such as CVP, can contribute essential nutrients like calcium, magnesium, and potassium, which are beneficial for health (41). Proteins are essential for growth and maintenance, providing vital amino acids necessary for various bodily functions (42). The protein content in date fruit spreads is significantly influenced by the addition of CVP, with values ranging from 2.21% in the control sample to 9.82% in CVP-supplemented samples. This enhancement in protein levels underscores the nutritional potential of integrating microalgae proteins into food products. This approach can also contribute to the development of innovative food products that cater to health-conscious consumers (43). The findings suggest that utilizing CVP in date spreads can improve their marketability and health benefits, aligning with the growing demand for high-protein food options (44). CVP can be integrated into various food products, including sauces and snacks, thereby expanding its market reach (38). The incorporation of CVP into date fruit spread formulas significantly enhances dietary fiber content, which is crucial for metabolic health. Table 2 indicates that the dietary fiber levels in these spreads ranged from 7.26 to 12.38, with CVP supplementation leading to a significant increase in fiber content, demonstrating its potential as a functional food ingredient. Dietary fiber is linked to the prevention of metabolic disorders such as obesity, type 2 diabetes, and cardiovascular diseases (45). Fiber promotes gut microbiota health, leading to the production of beneficial short-chain fatty acids (46). Increased fiber intake aids in preventing constipation and improving overall digestive health (47). CVP is rich in dietary fiber, contributing to the higher fiber levels in date spreads, with increases of 1.08 to 1.70 times compared to control samples. The use of CVP in food products aligns with trends in the food industry towards health-oriented formulations (46, 47). The correlation between higher levels of CVP and increased dietary fiber content highlights its potential as a functional food ingredient. This relationship not only enhances fiber intake but also contributes to various health benefits, including improved digestive health and reduced risk of chronic diseases (48). The incorporation of CVP into date spread formulas, significantly reduces carbohydrate content due to CVP’s inherently low carbohydrate profile. This reduction is evident, with carbohydrate levels decreasing from 51.93% in control samples to 46.82% with 10% CVP addition. The nutritional benefits of CVP extend beyond carbohydrate reduction, making it a valuable ingredient in food formulations. CVP is rich in proteins, lipids, vitamins, and minerals, contributing to its low carbohydrate content (38). The energy values of formulated date fruit spreads demonstrate a significant variation based on the inclusion of CVP. The addition of *Chlorella vulgaris* powder (CVP) to food formulations significantly impacts the energy content due to its low lipid levels. The control sample, which did not contain CVP, exhibited the highest energy value, while the incorporation of CVP led to a systematic decrease in energy values, quantified as reductions by factors of 1.03, 1.04, 1.11, 1.16, and 1.20 for 2, 4, 6, 8, and 10% CVP, respectively. This trend highlights the relationship between fat content and energy values in food products. Dates are rich in essential nutrients, including carbohydrates, dietary fiber, and minerals, which contribute to their overall energy content (8). The incorporation of CVP not only reduces energy values but may also enhance the nutritional profile by adding beneficial nutrients from CVP (Table 2). CVP is a rich source of protein and antioxidants, which can improve the overall nutritional quality of food products, such as processed cheese and snacks (40, 49). CVP incorporation has been shown to increase n-3 polyunsaturated fatty acids (PUFAs) while decreasing the n-6: n-3 ratio, thus improving the nutritional profile of the meat (50). Adding CVP to Karish cheese and processed cheese enhances their texture, antioxidant activity, and overall health benefits, making them functional foods (40, 51). Snacks with CVP exhibit lower glycemic indices and improved mineral bioaccessibility, contributing to healthier snack options (49). The mineral content in control date spread, including potassium, phosphorus, magnesium, calcium, and sodium, is significant, with values of 782.65 mg, 66.32 mg, 64.47 mg, 47.28 mg, and 4.40 mg per 100 g, respectively. These levels are beneficial as they meet daily human requirements for these essential minerals, contributing to overall health and nutrition. Dates are a nutrient-dense fruit, rich in essential minerals that contribute significantly to cardiovascular health, bone strength, and metabolic functions. They are particularly high in potassium, which aids in managing hypertension, while phosphorus supports bone health and energy production. Additionally, magnesium plays a crucial role in muscle and nerve function, and calcium contributes to bone density. However, while dates provide these benefits, relying solely on them for mineral intake may neglect the necessity of a varied diet (52). The iron, manganese, and zinc content in date fruit spread, measured at 0.56, 0.33, and 0.24 mg/kg respectively, appears to meet the nutritional requirements for these minerals. Dates are recognized for their rich mineral composition, which includes significant levels of iron and manganese, making them a beneficial dietary source for addressing deficiencies in these minerals (52). The incorporation of CVP into date fruit spread significantly enhances its mineral content, attributed to the high mineral richness of CVP. The results indicate that a 10% CVP addition results in a substantial increase in essential minerals compared to the control sample, highlighting the nutritional benefits of this microalga. *Chlorella vulgaris* is rich in minerals such as potassium, phosphorus, magnesium, calcium, sodium, iron, and zinc (38, 53). The results showed that the the content of potassium, phosphorus, magnesium, calcium, and sodium, iron and zinc in date spread with 10% CVP were about 1.16, 12.9, 2.31, 4.01, 3.29, 15.67 and 123.08 times higher, respectively, compared to the control without CVP addition. Dates themselves are a source of multiple minerals, including magnesium, potassium, and iron, which complement the mineral profile of CVP (Table 2). The combination of dates and CVP not only enhances mineral content but also provides health benefits, such as improved immune function and antioxidant properties (53). These findings suggest that integrating CVP into food products can significantly improve their nutritional value, making them more beneficial for consumers (Table 2).

**Table 2:** Chemical composition (g/100 g dry weight basis) and mineral content (mg/100 g) of date fruit spreads incorporated with different quantities of *Chlorella vulgaris* powder (CVP).

Components (g/100 g dry)	KDO0	KDO2	KDO4	KDO6	KDO8	KDO10
Moisture	13.98^a^ ± 0.39	13.75^b^ ± 0.45	13.00^b^ ± 0.89	12.50^c^ ± 0.54	12.19^d^ ± 0.67	12.0^e^ ± 0.22
Fat	36.65^a^ ± 1.45	34.17^b^ ± 1.38	32.00^c^ ± 1.43	30.12^d^ ± 1.23	27.80^e^ ± 1.04	25.05^f^ ± 1.37
Ash	1.95^e^ ± 0.09	2.86^d^ ± 0.43	3.28^c^ ± 0.54	4.71^b^ ± 0.31	5.15^a^ ± 0.21	5.93^a^ ± 0.23
Protein	2.21^f^ ± 0.08	3.91^e^ ± 0.02	5.23^d^ ± 0.16	6.84^c^ ± 0.31	7.98^b^ ± 0.32	9.82^a^ ± 0.45
Dietary fibers	7.26^f^ ± 0.79	7.89^e^ ± 0.97	8.20^d^ ± 0.29	9.96^c^ ± 0.45	11.97^b^ ± 0.96	12.38^a^ ± 0.89
Carbohydrate	51.93^a^ ± 1.49	51.17^b^ ± 1.48	48.71^c^ ± 1.89	48.37^cd^ ± 1.56	47.10^d^ ± 1.32	46.82^e^ ± 1.36
Energy (kcal/100 g)	546.42^a^ ± 5.98	527.85^b^ ± 7.22	503.67^c^ ± 6.97	491.92^d^ ± 5.81	470.52^e^ ± 5.29	452.01^f^ ± 5.38
Mineral content (mg/100 g)						
Na	4.40^f^ ± 0.22	6.42^e^ ± 0.94	8.44^d^ ± 0.84	10.46^c^ ± 0.87	12.48^b^ ± 0.79	14.50^a^ ± 0.54
Ca	47.28^f^ ± 2.90	75.78^e^ ± 2.31	104.28^d^ ± 3.98	132.78^c^ ± 4.88	161.28^b^ ± 3.87	189.78^a^ ± 4.89
K	782.65^f^ ± 6.92	808.59^e^ ± 7.92	834.53^d^ ± 7.97	860.47^c^ ± 6.89	886.41^b^ ± 5.74	912.35^a^ ± 5.09
Mg	64.47^f^ ± 2.09	81.49^e^ ± 2.85	98.51^d^ ± 3.76	115.53^c^ ± 2.94	132.55^b^ ± 2.89	149.57^a^ ± 3.92
P	66.32^f^ ± 2.13	224.33^e^ ± 8.30	382.35^d^ ± 5.98	540.37^c^ ± 4.90	698.38^b^ ± 5.09	856.40^a^ ± 4.76
Mn	0.33^f^ ± 0.04	0.43^e^ ± 0.06	0.52^d^ ± 0.06	0.61^c^ ± 0.04	0.71^b^ ± 0.08	0.80^a^ ± 0.19
Cu	0.15^f^ ± 0.07	1.12^e^ ± 0.05	2.08^d^ ± 0.07	3.05^c^ ± 0.76	4.02^b^ ± 0.06	4.99^a^ ± 0.34
Fe	0.56^f^ ± 0.94	2.20^e^ ± 0.09	3.85^d^ ± 0.76	5.49^c^ ± 0.44	7.14^b^ ± 0.87	8.78^a^ ± 0.65
Zn	0.24^f^ ± 0.02	6.10^e^ ± 0.32	11.96^d^ ± 0.84	17.82^c^ ± 0.98	23.68^b^ ± 0.43	29.54^a^ ± 0.97

Values are means ± SD of five determinations. Means in the same row with different letters are significantly different (*p* ≤ 0.05). KDO0: control date spread formula (without CVP), KDO2: date spread fortified with 2% CVP, KDO4: date spread fortified with 4% CVP, KDO6: date spread fortified with 6% CVP, KDO8: date spread fortified with 8% CVP and KDO10: date spread fortified with 10% CVP.

### 3.2 Amino acid contents (g/100 g protein) of date fruit spreads incorporated with different quantities of CVP

Table 3 shows the amino acid contents (g/100 g protein) of date fruit spreads incorporated with different quantities of CVP. The amino acid profile of control date fruit spread samples, reveals significant deficiencies in essential amino acids compared to the FAO/WHO pattern. The results showed that the control sample without CVP addition exhibited significant shortages in isoleucine, leucine, lysine, cystine, methionine, tyrosine, phenylalanine, and threonine, with lysine and total sulfur amino acids showing the highest deficiencies. This finding underscores the potential of *Chlorella vulgaris* to enhance the amino acid content of date fruit spreads. Lysine and total sulfur amino acids were the most deficient, which are critical for protein synthesis and metabolic functions (54). Incorporating CVP into date fruit spreads significantly enhances their nutritional profile, particularly in essential amino acids. The results indicate that a 10% addition of CVP can align the amino acid content of date spreads with FAO/WHO requirements, with the exception of lysine, which reaches 65.37% of the recommended levels (Table 3). This integration not only addresses amino acid deficiencies but also leverages the nutritional benefits of CVP. *Chlorella vulgaris* is rich in essential amino acids, particularly methionine and lysine, which could help mitigate deficiencies in date fruit spreads (53). In this regard, Wang et al.)2024(reported that the Incorporating *Chlorella vulgaris* into the diet can improve the overall amino acid profile, making it a valuable addition to plant-based foods. These findings highlight the importance of supplementing plant-based diets with high-quality protein sources like *Chlorella vulgaris* to address amino acid deficiencies (55). The predominant non-essential amino acids found in date fruit spreads were glutamic acid (18.77–26.74), aspartic acid (15.84–18.72), arginine (10.16–10.97), serine (4.17–4.26), proline (3.88–4.14), glycine (4.86–4.88), and alanine (4.53–4.92). The inclusion of these amino acids improves the nutritional profile of date fruit spreads, making them a good source of non-essential amino acids. The combination of these amino acids can improve the overall health advantages of date fruit spreads, making them ideal for functional food formulations (56). The incorporation of CVP into date fruit spreads significantly enhances their protein efficiency ratio (P-PER), indicating improved protein quality. The P-PER values for date fruit spreads incorporated with different quantities of CVP ranged from 2.24 to 2.36, with the highest values observed at 8.0 and 10.0% CVP supplementation. This suggests that microalgae like CVP can serve as effective protein sources in food formulations, aligning with the growing interest in sustainable protein alternatives. CVP is rich in proteins, with amino acid profiles superior to many plant proteins (57). Microalgae provide essential amino acids, vitamins, and minerals, enhancing the nutritional profile of food products (58). PER values below 1.5 indicate low-quality protein, while values above 2 suggest high-quality protein. This classification is essential for understanding dietary protein’s role in meeting essential amino acid (EAA) requirements and overall health (85). Therefore, all produced formulas are characterized by high protein quality. Incorporating high-quality proteins into diets can enhance EAA intake more effectively than increasing low-quality protein consumption (59).

**Table 3:** Amino acid contents (g/100 g protein) of date fruit spreads incorporated with different quantities of CVP.

Amino acid	KDO0	KDO2	KDO4	KDO6	KDO8	KDO10	FAO/WHO pattern (35)
Isoleucine	2.42	3.55	3.59	3.62	3.69	3.7	2.8
Leucine	6.28	6.42	6.52	6.55	6.76	6.85	6.6
Lysine	2.54	2.69	2.74	2.82	3	3.27	5.8
Cystine	0.7	1.46	1.49	1.55	1.62	1.68	
Methionine	0.86	0.9	0.9	0.93	0.95	0.99	
Total sulfur amino acids	1.56	2.36	2.39	2.49	2.57	2.67	2.5
Tyrosine	1.33	1.59	1.87	2.12	2,32	2.61	
Phenylalanine	3.69	4.7	4.8	4.91	4.92	4.95	
Total aromatic amino acids	5.02	6.29	6.67	7.03	7.24	7.56	6.3
Threonine	1.35	1.72	1.84	1.94	2.21	2.28	3.4
Valine	4.33	4.46	4.47	4.48	4.5	4.54	3.5
Tryptophan	0.96	2.45	2.48	2.52	2.55	2.95	1.1
Total essential amino acid	24.46	29.94	30.7	31.44	30.2	33.46	
Histidine	2.48	2.59	2.59	2.65	2.76	2.76	1.9
Arginine	10.16	10.3	10.48	10.55	11	10.97	
Aspartic acid	18.72	16.47	16.47	16.20	16.2	15.84	
Glutamic acid	26.74	22.86	21.90	21.19	21.20	18.77	
Serine	4.17	4.27	4.29	4.30	4.46	4.26	
Proline	3.88	4.01	4.01	4.03	4.27	4.14	
Glycine	4.86	4.86	4.87	4.87	4.97	4.88	
Alanine	4.53	4.7	4.69	4.77	4.94	4.92	
Total non-essential amino acid	75.54	70.06	69.3	68.56	69.8	66.54	
P-PER	2.24	2.28	2.29	2.29	2.35	2.36	

KDO0: control date spread formula (without CVP), KDO2: date spread fortified with 2% CVP, KDO4: date spread fortified with 4% CVP, KDO6: date spread fortified with 6% CVP, KDO8: date spread fortified with 8% CVP and KDO10: date spread fortified with 10% CVP.

### 3.3 Fatty acid compositions of date fruit spreads incorporated with different quantities of CVP

The Fatty acid compositions of date fruit spreads incorporated with different quantities of CVP are shown in Table 4. The incorporation of CVP into date fruit spreads significantly affects the fatty acid composition, particularly the levels of saturated fatty acids (SFA). As the percentage of CVP increases, the SFA content in the spreads rises, indicating a potential nutritional enhancement. The control sample of date fruit spreads contained 14.92% SFA, which increased to 24.42 and 26.79% with 8 and 10% CVP inclusion. This trend suggests that CVP is a rich source of saturated fatty acids, which can be beneficial for specific dietary needs (60). *Chlorella vulgaris* is known for its high lipid content, with studies showing significant levels of both saturated and unsaturated fatty acids, which can contribute to overall health (61). The incorporation of CVP into date spreads significantly enhances the levels of palmitic and stearic acids, as evidenced by a marked increase of 81.0 and 93.0%, respectively, compared to control samples without CVP. This finding underscores the nutritional benefits of integrating microalgae into food products. The fatty acid profile of *C. vulgaris* shows a predominance of C16-C18 fatty acids, indicating its potential as a dietary supplement (62). The incorporation of CVP into date fruit spreads significantly alters the fatty acid composition, particularly reducing mono-unsaturated fatty acids. This change is evident as the oleic acid content decreases from 79.3% in unfortified spreads to 67.10 and 64.02% with 8 and 10% CVP, respectively. The oleic acid concentration exhibited the same pattern. The incorporation of CVP into date fruit spreads significantly enhances their polyunsaturated fatty acid (PUFA) content, particularly linoleic (C18: 2 n-6) and linolenic (C18:3) acids. As the percentage of CVP increases, the PUFA levels rise correspondingly, indicating a beneficial nutritional enhancement. Date spreads with CVP showed a significant increase in PUFA content compared to the control, with increments of 17.6 to 59.16% for 2 to 10% CVP, respectively. Linoleic acid content rose from 5.08% in the control to 6.45% with 10% CVP, while linolenic acid increased from 0.7 to 2.75% at the same CVP level. The enhanced PUFA profile contributes to the health benefits associated with date consumption, such as improved cardiovascular health and anti-inflammatory effects, aligning with findings on the nutritional value of dates (52). The fatty acids in CVP oil exhibit antioxidant and anti-inflammatory activities, which can mitigate oxidative stress and inflammation (61). The diverse fatty acid profile of CVP suggests potential applications in nutraceuticals and functional foods, enhancing overall health outcomes (53).

**Table 4:** Fatty acid compositions of date fruit spreads incorporated with different quantities of CVP.

Fatty acids	KDO0	KDO2	KDO4	KDO6	KDO8	KDO10
Myristic(C14:0)	ND	ND	0.22^d^ ± 0.07	0.28^c^ ± 0.07	0.38^b^ ± 0.09	0.46^a^ ± 0.09
Palmitic(C16:0)	12.16^f^ ± 0.56	14.13^e^ ± 0.57	16.15^d^ ± 0.87	18.07^c^ ± 0.89	20.03^b^ ± 0.97	22.01^a^ ± 0.09
Stearic (C18:0)	1.90^f^ ± 0.04	2.26^e^ ± 0.09	2.61^d^ ± 0.72	2.97^c^ ± 0.43	3.32^b^ ± 0.09	3.67^a^ ± 0.04
Arachidic (20:0)	0.86^a^ ± 0.04	0.82^b^ ± 0.08	0.77^c^ ± 0.03	0.73^d^ ± 0.09	0.69^e^ ± 0.02	0.65^f^ ± 0.02
Oleic (C18:1)	79.3^a^ ± 0.98	76.00^b^ ± 1.74	73.01^c^ ± 0.89	70.03^d^ ± 1.92	67.10^e^ ± 1.87	64.02^f^ ± 1.67
Linoleic (C18:2)	5.08^f^ ± 0.34	5.59^e^ ± 0.67	5.77^d^ ± 0.76	5.99^c^ ± 0.70	6.16^b^ ± 0.74	6.45^a^ ± 0.76
Linolenic (C18:3)	0.70^f^ ± 0.06	1.21^e^ ± 0.06	1.52^d^ ± 0.05	1.93^c^ ± 0.05	2.34^b^ ± 0.07	2.75^a^ ± 0.08
∑SFA	14.92^f^ ± 0.54	17.21^e^ ± 0.34	19.75^d^ ± 0.78	22.05^c^ ± 0.87	24.42^b^ ± 0.78	26.79^a^ ± 0.43
∑MUFA	79.30^a^ ± 1.34	76.00^b^ ± 1.83	73.01^c^ ± 1.45	70.03^d^ ± 1.56	67.10^e^ ± 1.92	64.02^f^ ± 1.87
∑PUFA	5.78^f^ ± 0.67	6.80^e^ ± 0.67	7.29^d^ ± 0.79	7.92^c^ ± 0.56	8.50^b^ ± 0.82	9.20^a^ ± 0.93

Values are means ± SD of 2 determinations. Means in the same row with different letters are significantly different (*p* ≤ 0.05). KDO0: control date spread formula (without CVP), KDO2: date spread fortified with 2% CVP, KDO4: date spread fortified with 4% CVP, KDO6: date spread fortified with 6% CVP, KDO8: date spread fortified with 8% CVP and KDO10: date spread fortified with 10% CVP. PUFA Polyunsaturated Fatty Acids = UFA Unsaturated fatty acids MUFA Mono Unsaturated fatty acids. SFA Saturated fatty acids.

### 3.4 Total phenolics, total flavonoids and DPPH radical scavenging activity % of date fruit spreads incorporated with different quantities of CVP

Total phenolics, total flavonoids and DPPH radical scavenging activity % of date fruit spreads incorporated with different quantities of CVP are shown in Table 5. Total phenolic content (mg GAE/g dry weight) of date spread formulas ranged from 54.11 to 69.50. The combination of date fruits with extra virgin olive oil (EVOO), light roasted coffee, and unsweetened cocoa powder can significantly enhance the total phenolic compounds in date fruit spread samples. This enhancement is attributed to the synergistic effects of the individual components, each contributing unique bioactive compounds and antioxidant properties. Date fruits are rich in bioactive compounds, including phenolics, which possess antioxidant, anti-inflammatory, and antimicrobial properties (63). EVOO is known for its high concentration of biophenols, which can act as natural antioxidants, enhancing the stability and health benefits of food products (64). The combination of these ingredients not only boosts the antioxidant capacity but also improves the sensory attributes of the spread, appealing to health-conscious consumers (65). The incorporation of CVP into date fruit spreads significantly enhances their total phenolic content, which is vital for improving the nutritional profile of food products. The total phenolic content in date spreads increased from 54.11 mg GAE/g in the control sample to 69.50 mg GAE/g with 10% CVP fortification, demonstrating the antioxidant potential of CVP due to its rich phenolic compounds (Table 5). This enhancement is crucial as phenolic compounds are known for their health benefits, including antioxidant, anti-inflammatory, and immunomodulatory effects (86). The total flavonoid content in date spread formulas ranged from 16.90 to 29.95 mg CE/g dry weight. Control date spread formula had 16.90 mg CE/g dry weight. This value highlights the significant contribution of its ingredients, including date fruits, olive oil, roasted coffee, and cocoa powder. These components are known for their rich bioactive compounds, which enhance the flavonoid content in the final product. Date fruits contain high levels of flavonoids, with studies showing total flavonoid content varying from 46.59 to 111.80 mg QE/100 g dry weight (66). Flavonoids, abundant in dates (*Phoenix dactylifera*), contribute significantly to their health-promoting effects, particularly through their anti-inflammatory and antioxidant activities. Research indicates that various date cultivars possess high concentrations of flavonoids and other phenolic compounds, which enhance their functional food status (67). Cocoa powder is a rich in polyphenols, contributing to the overall antioxidant capacity and enhancing the nutritional profile of the spread (68). Roasted coffee is known for its high antioxidant properties, further boosting the flavonoid content (69). The incorporation of CVP into date spread formulations significantly enhances the total flavonoid content, with increases observed from 16.90 mg CE/g dry weight to as high as 29.95 mg CE/g dry weight at 10% CVP supplementation. This improvement in flavonoid levels is indicative of the bioactive compounds present in CVP, which contribute to the nutritional and functional properties of food products (Table 5). CVP is rich in various bioactive compounds, including carotenoids and chlorophyll, which contribute to its antioxidant activity (53). The incorporation of CVP not only boosts flavonoid content but also adds essential nutrients, making the date spread a functional food (70). Flavonoids are known for their antioxidant properties, which can enhance the health benefits of food products (71). The DPPH radical scavenging activity of date spread formulas, ranged from 55.90 to 68.00%. Control date spread formula had DPPH radical scavenging activity % of 55.90%, highlights the antioxidant potential of its ingredients, particularly date fruits, olive oil, roasted coffee, and cocoa powder. This activity is attributed to the synergistic effects of these components, which enhance their individual antioxidant properties. Date fruits (*Phoenix dactylifera*) are rich in phenolic compounds, which contribute significantly to their antioxidant capacity. Studies have shown that different varieties of dates contain varying levels of phenolics, with some exhibiting high concentrations that correlate with antioxidant activity (67, 72). The DPPH assay indicates that date extracts can effectively scavenge free radicals, with reported activities ranging from 54.43 to 80.89% (72, 73). The combination of antioxidants from different sources, such as olive oil and cocoa powder, can lead to enhanced scavenging activity. This synergism can significantly improve the overall antioxidant effectiveness of food products (74). The incorporation of CVP into date fruit spread formulas significantly enhances the DPPH radical scavenging activities, demonstrating its potential as a functional food ingredient. The observed increase in antioxidant activity, from 55.90 to 58.85, 60.10, 62.15, 65.10 and 68.00 for those samples fortified with 2, 4, 6, 8, and 10% of CVP, underscores the microalga’s rich bioactive composition, which includes antioxidants that combat oxidative stress. CVP is rich in bioactive compounds such as carotenoids, polyphenols, and tocopherols, which are known for their antioxidant properties (75). Studies indicate that microalgae like *C. vulgaris* can effectively scavenge free radicals, thereby reducing oxidative stress and its associated health risks (53). In this regard Wang et al. (38) reported that *C. vulgaris* is a nutrient-dense food source, providing high-quality proteins, essential vitamins, and minerals, which contribute to overall health and well-being. The incorporation of CVP not only enhances antioxidant activity but also improves the nutritional profile of food products like date spreads (38).

**Table 5:** Total phenolics, total flavonoids and DPPH radical scavenging activity % of date fruit spreads incorporated with different quantities of CVP.

Parameters	KDO0	KDO2	KDO4	KDO6	KDO8	KDO10
Total phenolics (mg GAE/g dry weight)	54.11^e^ ± 2.45	56.90^d^ ± 3.76	59.00^d^ ± 3.72	62.10^c^ ± 3.76	66.60^b^ ± 3.92	69.50^a^ ± 2.54
Total flavonoid mg catechin equivalents (CE)/g dry weight	16.90^f^ ± 1.34	19.17^e^ ± 1.26	22.70^d^ ± 1.02	25.53^c^ ± 1.34	27.98^b^ ± 2.71	29.95^a^ ± 1.63
DPPH radical scavenging activity %	55.90^f^ ± 2.45	58.85^e^ ± 2.54	60.10^d^ ± 2.46	62.15^c^ ± 2.78	65.10^b^ ± 2.90	68.00^a^ ± 2.47

Values are means ± standard deviation (SD) of five determinations. Means in the same row with different letters are significantly different (*p* ≤ 0.05). KDO0: control date spread formula (without CVP), KDO2: date spread fortified with 2% CVP, KDO4: date spread fortified with 4% CVP, KDO6: date spread fortified with 6% CVP, KDO8: date spread fortified with 8% CVP and KDO10: date spread fortified with 10% CVP.

### 3.5 Sensory properties of date fruit spreads incorporated with different quantities of CVP

The sensory properties of food, such as taste, texture, odor, and appearance, are crucial for consumer acceptability. The sensory properties of date fruit spreads incorporated with different quantities of CVP are presented in Table 6. In the case of date fruit spreads with varying levels of CVP, appearance scores ranged from 8.00 to 9.15, indicating a generally favorable perception. Statistically, no significant differences were found in appearance between control samples and those with 2 and 4% CVP, while the 10% CVP sample received the lowest score. This highlights the importance of sensory evaluation in food product development. The appearance of food significantly influences consumer choices, with higher scores correlating to better acceptability (76). The incorporation of CVP into date fruit spreads significantly influences taste scores, which are critical for consumer acceptability. The highest scores were observed at lower supplementation levels (2 and 4% CVP), indicating these levels enhance flavor without overpowering the product, while higher levels (6–10%) resulted in a marked decline in taste ratings, with the lowest score recorded at 10% CVP. A taste score of 7.00 at 10% CVP suggests that excessive supplementation can negatively impact consumer perception. This trend underscores the importance of balancing ingredient proportions to optimize sensory attributes. The flavor score of date fruit spread samples indicates a significant relationship between flavor enhancement and the addition of *Chlorella vulgaris* powder (CVP). The highest flavor scores (9.50 and 9.55) were achieved with 2 and 4% CVP, respectively, indicating a sweet spot for flavor enhancement. The addition of CVP improved flavor scores significantly, suggesting that moderate fortification can enhance product appeal. Conversely, higher concentrations led to a decline in flavor quality. The addition of 10% CVP resulted in the lowest flavor score (7.30), highlighting the potential for over-fortification to detract from flavor quality. This trend underscores the importance of flavor in consumer acceptance, as it is a critical quality attribute that influences purchasing behavior (77). The texture of food products significantly influences consumer acceptance, as evidenced by the findings regarding date spreads supplemented with varying concentrations of CVP. While no significant differences were noted in texture scores between control and samples with 2 and 4% CVP, higher concentrations (6–10%) led to marked declines in texture quality, with the lowest score (6.50) recorded at 10% CVP. This highlights the delicate balance required in ingredient formulation to maintain desirable textural properties. This aligns with findings that suggest optimal ingredient levels are crucial for maintaining desirable sensory attributes (78). Color is a critical factor in consumer acceptance, as it influences perceived quality and freshness (79). The relationship between color and consumer acceptance in date spreads is significant, particularly when considering the effects of color additives like CVP. The results indicate that while lower concentrations of CVP (2–4%) do not significantly alter color scores, suggesting these levels maintain acceptable color quality. In contrast, higher CVP concentrations (6–10%) result in significant decreases in color scores, indicating a threshold beyond which color quality deteriorates This highlights the importance of maintaining optimal color attributes for product acceptance. The observation of the lowest color value (6.00) in date fruit samples fortified with 10% CVP highlights the impact of fortification on the colorimetric properties of dates. This finding suggests that the addition of certain compounds can significantly alter the visual characteristics of date fruits, which is crucial for consumer acceptance and marketability. The overall acceptability of food products is crucial for consumer acceptance, particularly when incorporating novel ingredients like *Chlorella vulgaris* powder (CVP). The results indicate that while lower concentrations of CVP (2–4%) do not significantly affect acceptability, higher concentrations (6–10%) lead to significant declines in consumer ratings. Specifically, date spreads fortified with 8 and 10% CVP received the lowest scores (7.75 and 6.96), highlighting a threshold beyond which acceptability diminishes. Similar trends are observed in other studies, were ingredient modifications impact sensory attributes and overall acceptability. For instance, the addition of Chlorella sp. in Puto showed that concentrations above 2% negatively affected taste and acceptability (80). Conversely, some studies suggest that innovative ingredients can enhance acceptability when marketed effectively, as seen with millet-based products that received positive consumer feedback despite their unconventional nature (81, 82). This indicates that consumer education and marketing strategies play a vital role in acceptance.

**Table 6:** Sensory properties of date fruit spreads incorporated with different quantities of CVP.

Parameter	KDO0	KDO2	KDO4	KDO6	KDO8	KDO10
Appearance	9.15^a^ ± 0.45	9.17^a^ ± 0.45	9.17^a^ ± 0.54	9.00^b^ ± 0.76	8.25^c^ ± 0.38	8.00^cd^ ± 0.48
Taste	9.35^b^ ± 0.34	9.40^ab^ ± 0.65	9.50^a^ ± 0.67	8.00^c^ ± 0.89	7.50^d^ ± 0.58	7.00^e^ ± 0.72
Flavor	9.40^b^ ± 0.22	9.50^a^ ± 0.73	9.55^a^ ± 0.83	8.50^c^ ± 0.87	8.00^d^ ± 0.37	7.30^e^ ± 0.33
Texture	9.10^a^ ± 0.65	9.10^a^ ± 0.89	9.10^a^ ± 0.57	8.50^b^ ± 0.46	7.00^c^ ± 0.95	6.50^d^ ± 0.49
Color	9.45^a^ ± 0.68	9.50^a^ ± 0.94	9.50^a^ ± 0.98	8.50^b^ ± 0.75	8.00^c^ ± 0.92	6.00^d^ ± 0.12
Overall acceptability	9.27^a^ ± 0.37	9.32^a^ ± 0.98	9.36^a^ ± 0.73	8.62^b^ ± 0.49	7.75^c^ ± 0.64	6.96^d^ ± 0.93

Data are expressed as the mean ± standard deviation. Means in the same row with different letters are significantly different (*p* ≤ 0.05). KDO0: control date spread formula (without CVP), KDO2: date spread fortified with 2% CVP, KDO4: date spread fortified with 4% CVP, KDO6: date spread fortified with 6% CVP, KDO8: date spread fortified with 8% CVP and KDO10: date spread fortified with 10% CVP.

## 4 Conclusion

The substitution of extra virgin olive oil (EVOO) with *Chlorella vulgaris* powder (CVP) in date-based spreads significantly alters their nutritional profile and sensory characteristics. The results indicate that formulations with CVP not only reduce fat content but also enhance protein and mineral levels, making them potentially healthier alternatives. Protein content increases from 2.21% in control to 9.82% with 10% CVP. DPPH radical scavenging activity improves, indicating that CVP enhances the spread’s antioxidant properties. Lower concentrations of CVP (2–4%) maintain acceptability, while higher concentrations (6–10%) lead to significant declines in sensory appeal.

## Data Availability

The original contributions presented in the study are included in the article/supplementary material, further inquiries can be directed to the corresponding author.
